# Non-Coding RNA Databases in Cardiovascular Research

**DOI:** 10.3390/ncrna6030035

**Published:** 2020-09-02

**Authors:** Deepak Balamurali, Monika Stoll

**Affiliations:** 1 Genetic Epidemiology and Statistical Genetics, Department of Biochemistry, CARIM School for Cardiovascular Diseases, Maastricht University, 6229 ER Maastricht, The Netherlands; d.balamurali@maastrichtuniversity.nl; 2 Institute of Human Genetics, Genetic Epidemiology, University of Muenster, 48149 Muenster, Germany; 3 Maastricht Center for Systems Biology (MaCSBio), Maastricht University, 6229 ER Maastricht, The Netherlands

**Keywords:** database, ncRNA, cardiovascular diseases, non-coding

## Abstract

Cardiovascular diseases (CVDs) are of multifactorial origin and can be attributed to several genetic and environmental components. CVDs are the leading cause of mortality worldwide and they primarily damage the heart and the vascular system. Non-coding RNA (ncRNA) refers to functional RNA molecules, which have been transcribed into DNA but do not further get translated into proteins. Recent transcriptomic studies have identified the presence of thousands of ncRNA molecules across species. In humans, less than 2% of the total genome represents the protein-coding genes. While the role of many ncRNAs is yet to be ascertained, some long non-coding RNAs (lncRNAs) and microRNAs (miRNAs) have been associated with disease progression, serving as useful diagnostic and prognostic biomarkers. A plethora of data repositories specialized in ncRNAs have been developed over the years using publicly available high-throughput data from next-generation sequencing and other approaches, that cover various facets of ncRNA research like basic and functional annotation, expressional profile, structural and molecular changes, and interaction with other biomolecules. Here, we provide a compendium of the current ncRNA databases relevant to cardiovascular research.

## 1. Introduction

Cardiovascular diseases (CVD) are ailments of the heart and the circulatory system and include a variety of highly prevalent disorders such as coronary artery disease (CAD), atherosclerosis (AS), atrial fibrillation (AF), ischemic heart failure (IHF), and congenital heart disease (CHD). Although CVDs are multifactorial in their origin, the most common causes are genetic and environmental changes. CVDs are the prime trigger of premature mortality worldwide, accounting for over 17.9 million deaths, as of 2016 [1]. In Europe, there were 11.3 million new cases of CVDs and 3.9 million deaths due to CVDs during 2015, with estimated costs of over 210 billion euros to the European Union (EU) [2]. The socio-economic burden due to CVDs is therefore constantly increasing, placing huge stress on the immediate need to develop novel diagnostic and therapeutic strategies for the control, treatment, and management of CVDs.

Non-coding RNAs (ncRNAs) are a type of RNA molecule, which are not translated into proteins. In humans, less than 2% of the genome correlates to the coding region, despite a transcription rate as high as even 93% [3,4,5]. More recently, the exponential increase in next-generation sequencing and other similar high-throughput methods has made available a plethora of information on ncRNAs. These ncRNAs can be further distinguished into housekeeping ncRNAs and regulatory ncRNAs. Housekeeping ncRNAs mainly are comprised of the ribosomal RNA (rRNA), transfer RNA (tRNA), small nuclear RNA (snRNA), and small nucleolar RNA (snoRNA). These molecules are involved in many key cellular processes. Regulatory ncRNAs are comprised of long and small non-coding RNAs. The former group consists of long non-coding RNAs (lncRNAs), circular RNAs (circRNAs), antisense RNAs (asRNAs), and enhancer RNAs (eRNAs) that are 200nts or longer, while the latter comprises smaller molecules such as microRNAs (miRNAs), small interfering RNAs (siRNAs), and piwi-associated RNAs (piRNAs) which are about 20nts in length.

Increasing studies have indicated ncRNAs to have a role in the progression of cancers, cardiovascular diseases, and other complex disorders, serving as a regulator of numerous cellular processes [6]. Additionally, there has been a spurt in the number of projects working on various interaction mechanisms in ncRNAs, such as lncRNA–lncRNA interactions, lncRNA–miRNA interactions, and lncRNA–miRNA–mRNA competing endogenous RNA interactions (ceRNA) to name a few [7,8]. The availability of open reading frames (ORFs) in some lncRNAs facilitates their translation into proteins and this further increases the ambiguity between coding and non-coding genes [9]. Furthermore, the use of high-throughput sequencing technologies has helped identify the potential role of many ncRNAs both in the pathogenesis of CVDs as well as in their role as therapeutic target molecules [10,11,12]. For instance, the lncRNA MHRT in humans has been associated with cardiac hypertrophy by regulation of the miR-145a-5p/KLF4/myocardin axis [13]. The lncRNA has also found to inhibit the apoptosis of cardiomyocytes, indicating that it might serve as a putative diagnostic and prognostic biomarker for chronic heart failure [14,15]. Chronic heart failure patients with lower expression levels of lncRNA MHRT had worse survival conditions compared to patients with higher expression levels of lncRNA MHRT [15].

A key characteristic in support of ncRNAs is that they are easily detectable biomarkers in body fluids (blood, saliva, cerebrospinal fluid (CSF), etc.) promoting the use of minimally-invasive techniques such as liquid biopsies [14,16]. A recent review by Meier et al. has stressed the potential role of circulating biomarkers, particularly circulating white blood cells (WBCs), in the diagnosis of heart failure (HF) [17]. With multiple studies showing that chronic inflammation levels play a critical role in the initiation and progression of HF, measuring WBC levels would help specifically diagnose the disease in its early stages [18,19,20]. There is thus a growing interest in deciphering the role of non-coding RNAs in disease causality, progression, and therapeutic targeting. To satisfy this growing interest, over 200 databases have been created. However, only a handful of these repositories are maintained systematically and being updated periodically. Previous reviews have listed lncRNA databases associated with CVDs, the most recent of these being in 2016 [21,22,23,24]. In this age of fast-paced genomic discoveries, it is important to periodically assess the repositories for updates and extended functionalities. All the databases reviewed here have been updated at least once since January 2018 (Figure 2). We have used some prominent ncRNAs related to CVDs as examples to investigate the salient features in each of these repositories.

## 2. Noncoding RNA Databases

**Figure 1:**
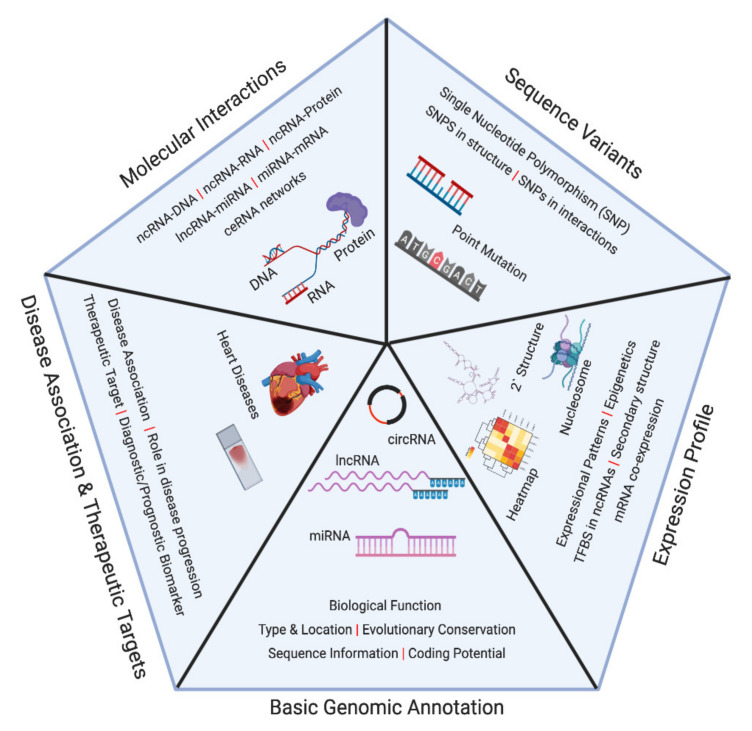
Segregation of information curated on various non-coding RNA databases. The available data have been grouped into five categories: basic genomic annotation, expression profile, molecular interactions, Disease association and therapeutic targets, and sequence variants. Databases for each of these kinds of information are listed in Figure 2.

**Figure 2:**
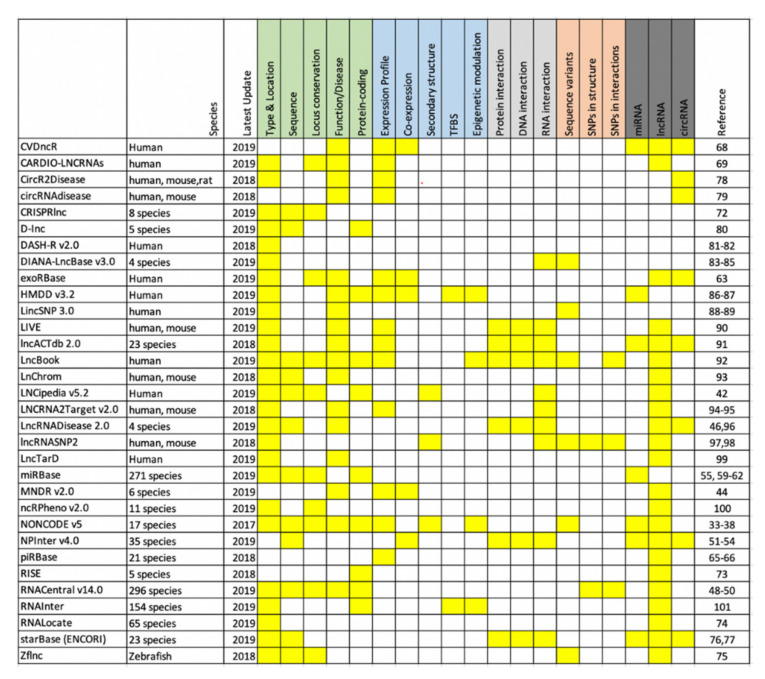
Overview of current ncRNA databases: The available databases have been grouped into five categories: basic genomic annotation (green), expression profile (blue), molecular interactions (light grey), Disease association (orange) and ncRNA type (dark grey). The corresponding boxes are shaded in yellow if the details are present in the respective databases [78,79,80,81,82,83,84,85,86,87,88,89,90,91,92,93,94,95,96,97,98,99,100,101].

The following ncRNAs were used to test the properties of the respective noncoding RNA databases. All of these ncRNAs are relevant to cardiovascular diseases and are detailed in the following section. Myosin heavy chain associated RNA transcript (MHRT) is a gene encoding a spliced form of a non-coding RNA, acting as a cardioprotective agent in the heart. This lncRNA is conserved in nature, seen also in mice (Mhrt), where they have been shown to serve as a potential biomarker for cardiac hypertrophy and heart failure [13,14,15,25,26]. CDKN2B-AS1 or ANRIL (antisense non-coding RNA in the INK4 locus) has been previously implicated for its role in post-ischemic angiogenesis through the Akt phosphorylation. Overexpression of ANRIL promoted angiogenesis and improved cardiac function significantly [27]. miR-132 has been extensively studied as a putative therapeutic biomarker, due to its role in cardiac hypertrophy and heart failure, through its effect on the pro-hypertrophic calcineurin/NFAT pathway [28,29]. The lncRNA encoded by splicing of the gene RP4-758J18.2 has previously been shown to play a role in the progression of systemic lupus erythematosus (SLE) through its interaction with CCNL2 gene [30]. The locus, chr.1p36 also frequent genomic rearrangements, leading to conditions like craniosynostosis and others, which in turn affect the normal functioning of the heart [31,32]. We checked the non-coding RNA databases for information on sequence annotation, genome build and evolutionary conservation, functional significance, and other parameters for these examples and have presented the results below (Figure 1 and Figure 2).

### 2.1. NONCODE V5

NONCODE was originally published in 2005, serving as the first unified resource on ncRNAs [33]. Since then, the database has been updated constantly [34,35,36,37], with the latest version being NONCODE v5, integrating data from 548,640 transcripts across 17 different species including 172,216 human lncRNA transcripts for 96,308 lncRNA genes [38]. NONCODE serves as a unified database containing information on all non-coding RNAs (excluding tRNAs and rRNAs) with a special focus on lncRNAs. The most recent update of this database includes salient features such as human lncRNA-disease relationships, SNP-lncRNA-disease relationships, and human exosomal lncRNA expression profiles. The data has been curated from extensive literature mining, as well as from specialized databases like GenBank, Ensembl, RefSeq, lncRNAdb, and Lncipedia [39,40,41,42]. The database has also added options for RNA secondary structure prediction with aid of RNAex [43]. NONCODE follows an exclusive system of nomenclature wherein a three-letter code for each species is prefixed by “NON” and suffixed by G/T depending on whether it is a gene/transcript, followed by six sequential numbers and a version number wherever applicable. For MHRT (NONHSAG068639.2), we were able to locate five transcript isoforms on chromosome 14, which are highly expressed in the thyroid, skeletal muscles, breast, and testes tissues. Despite the known association of MHRT with cardiac hypertrophy and other disorders, we did not find such information on NONCODE. In the case of ANRIL (NONHSAG101229.2), the search retrieved details of 53 unique transcripts that code for this lncRNA, as well as details of its tissue specific expression profile from Human BodyMap data. The search also displays a detailed table with disease association for ANRIL, rightly linking it with AS, CHD, CAD, and many types of cancers. Links to the original publication, and the source database are also provided [44,45,46]. The search also retrieved a comprehensive list of mutations reported in ANRIL till date, with detailed information on its incidence and GWAS traits [47].

### 2.2. RNAcentral v14.0

RNAcentral is an exhaustive ncRNA database containing a collection of different types of ncRNA sequences from a wide range of species [48]. Launched in 2014 and subsequently updated many times, RNAcentral curates data from over 40 databases to provide information on the various attributes of ncRNAs [49,50]. This database is among the most consistently updated resources, with the latest versions including a new sequence viewer, autocomplete capabilities on the search bar, structured snippets as part of search results as well as a JSON-based submission pipeline. RNAcentral is also among the primary choices of researchers for reference ncRNA datasets to be used in analyses. A search for MHRT (URS00007E4CE3_9606) provided a detailed page with five genome annotations, sequence and genome locations, and a list of all publications featuring the lncRNA MHRT to-date. Similarly, a search for miR-132 retrieved a detailed list of 205 sequences from over 50 organisms, indicating that the miRNA has been conserved across evolution. The results also displayed links to its annotation in 8 other databases, as well as 40+ manuscripts on miR-132 published so far.

### 2.3. NPInter v4.0

NPInter is a repository of experimentally-verified functional interactions between the ncRNAs and other biomolecules like genomic DNA, RNA, and protein. NPInter was first published in 2006 and subsequently updated periodically [51,52,53]. The latest version of the database is NPInter v4.0, published earlier this year and contains interaction data from more than 35 species, including 877,002 interactions reported in humans [54]. The interactions are manually curated from peer-reviewed literature and annotated against reference databases like NONCODE, miRbase, and UniProt [38,55,56]. NPInter v4.0 has seen the addition of interactions of circRNAs, interactions between lncRNAs and the genome and the integration of disease association with the interaction function. The interactions are broadly classified into binding, regulatory, and expression correlation. Apart from systematic literature mining, the database also curates interaction information from CLIP-seq, AGO CLIP-seq, and ChIRP-seq datasets that are publicly available. For ANRIL (NONHSAG051899), the database search retrieved 62 interaction hits, with details of the interaction partner, a brief description of the experimental design, and the publications’ PubMed IDs. The search also provided disease annotation for CDKN2B-AS1, with 63 results retrieved from MNDR database, with associated PubMed IDs wherever available. There was no information available on the database for MHRT and cANRIL.

### 2.4. miRbase v22

miRbase is a repository of miRNA sequences and annotations, originally published in 2002 as the microRNA registry [57,58] and subsequently updated regularly, with the most recent publication being in 2019 [55,59,60,61,62]. v22 contains microRNA sequences from 271 species accounting for 38,589 hairpin precursors and 48,860 mature miRNAs. A search for miR-132 on the database retrieves a detailed result, which includes external links to other reference databases, a list of 285 open-access publications which mention miR-132 as well as details of 157 deep sequencing experiments across different species, which explore the selected miRNA.

### 2.5. exoRBase

exoRBase is a repository of lncRNAs, circRNAs and mRNAs from RNA-seq analysis of human exosomes [63]. The database integrates information from normal and diseased patient samples and visualizes the changes in expression profile. Experimental validations from published datasets have also been included in this project. The current curation of the database holds 3914 circRNAs and 1628 lncRNAs with a known association with coronary heart disease (CHD). There were no reports of miR-132 and MHRT in the repository. However, a search for CDKN2B-AS1 on the database retrieved its expression profile across 92 samples, along with information about related circRNA (cANRIL) [64].

### 2.6. piRBase

piRBase is an exclusive database for all information about PIWI-interacting RNAs, a small subset which is highly expressed in germline tissues and is being increasingly shown to have a role in epigenetic and post-transcriptional modulations. The database was originally launched in 2014 and has subsequently been updated in 2018, covering 21 species and 173 million piRNA [65,66]. The revised version also provides aggregated information on piRNA targets and potential disease associations. With the exception of piRNABank [67] which has not been updated since 2008, piRBase remains the only dedicated resource for piRNA research.

## 3. Noncoding RNA Databases in Cardiovascular Diseases

### 3.1. CVDncR

CVDncR is a database of manually collected data of non-coding RNA, especially microRNA, long non-coding RNA and circular RNA, related to cardiovascular diseases [68]. The database includes 23 cardiovascular diseases at the moment. It also provides information on the applications of the ncRNA (diagnosis, prognosis, or treatment), expression profile and validation techniques, along with links to publications on the selected candidate. A search for MHRT retrieved three results, relating to its association with acute myocardial infarction, hypertension and heart failure. Furthermore, each result provided detailed information on the ncRNA’s biomarker status, expression pattern, validation data and sample details, as well as information to publications on the ncRNA. Nine results of miR-132 association with AF and HF among others was also available on the database, with detailed annotation.

### 3.2. CARDIO-LNCRNAs

This unique database provides a landscape of the transcription in human heart tissue [69]. RNA-seq datasets were retrieved from public repositories such as GEO and ArrayExpress [70,71]. The lncRNAs were classified as heart-specific (HS), heart-enriched (HES), heart-enhanced (HEH), and expressed in all tissues (EIA) based on their expression profile. This atlas also covers information on the variance in expression between diseased and normal heart tissue, SNPs related to CVDs and the conservation of lncRNAs in different developmental stages. Upon searching for the lncRNA of the gene RP4-758J18.2, we are presented with detailed information about its expression profile across 156 heart tissue samples, the expression across different developmental stages and the comparative expression in other tissues.

## 4. Other Resources

Apart from the resources above, there are other unique databases such as CRISPRlnc, RISE, RNAlocate, and MNDR which serve specific requirements. CRISPRlnc is a manually curated database of validated CRISPR/Cas9 sgRNAs for lncRNAs from eight species. The database comprised of 2100+ sgRNAs for 300+ lncRNA sequences [72]. RISE is a comprehensive repository of RNA-RNA interactions. The interactions mainly come from recent transcriptome-wide sequencing-based experiments and targeted studies that also include interactions aggregated from other primary databases and publications [73]. RNAlocate holds close to 200,000 RNA-associated subcellular localization entries with experimental and predicted evidence encompassing more than 40 subcellular localizations from 65 species [74]. Mammalian ncRNA-disease repository (MNDR) is an accumulation of validated and predicted ncRNA-disease associations drawn together from manual literature curation. The current update to this database covers over 260,000 entries from six species, associating the ncRNAs with 1416 diseases [44]. A search for “Heart Failure” yielded 254 results, each with additional information on MeSH, related target genes and a confidence score, based on whether the result had been validated by a strong or weak experimental system. There are also species-specific databases such as Zflnc, that have been created to assist researchers working on zebrafish. Zflnc is a comprehensive and well-annotated database for lncRNAs in Zebrafish, providing information on sequence conservation, pathway, and OMIM annotation and also expression profiling [75].

ENCORI (The Encyclopedia of RNA Interactomes), previously known as starBase, is also a useful resource which has now been updated to include over 7 million interactions between RNA subtypes, mined from publicly available big-data experiments [76,77]. The database covers information from 23 species and holds close to 20,000 functional annotation terms across 15 categories. Apart from these resources, there exist other databases (ANGIOGENES, C-It-Loci and circBase, to name a few) that have been included in previous reviews but have been excluded here. ANGIOGENES is a knowledgebase of angiogenesis, to explore and compare the expression profiles of transcripts in endothelial cells. C-It_Loci provides information on tissue-enriched loci that can be further screened in-silico to obtain anticipated positional and sequence conservation profiles. Though these databases have salient features, their content has not been updated in more than 24 months and have therefore not been considered here. A detailed list of all ncRNA resources has been provided in Supplementary Table S1.

## 5. Future Perspectives

Despite the existence of numerous resources in support of noncoding RNA research in cardiovascular genetics, there remain questions that are yet to be decoded. Some of the areas, which will require future attention include the evolutionary conservation of lncRNAs in heart tissue and lncRNA-circRNA associations among others. The recent advances in the field of high-throughput sequencing have led to sophisticated methods for RNA detection, such as total RNA sequencing and single-cell sequencing. This, in turn, has drastically increased the discovery rate of ncRNAs, particularly lncRNAs and circRNAs in recent years. Keeping in mind recent findings that these ncRNAs have an extended role in disease initiation, progression, and general pathophysiology, RNA molecules have now emerged as a strong therapeutic candidate against CVDs and such databases will prove a pivotal resource for researchers working in this domain.

## Supplementary Materials

The following are available online at https://www.mdpi.com/2311-553X/6/3/35/s1, Table S1: List of ncRNA databases with additional information on the database, manuscript, and year of publication.

## Abbreviations

AF	Atrial Fibrillation
AS	Atherosclerosis
asRNA	Antisense RNA
CAD	Coronary Artery Disease
cANRIL	Circular Antisense Noncoding RNA in the INK4 Locus
CCNL2	Cyclin L2
ceRNA	Competing endogenous RNA
CHD	Congenital Heart Disease
circRNA	Circular RNA
CLIP-Seq	Cross-linking Immunoprecipitation sequencing
ChiRP-Seq	Chromatin Isolation by RNA purification
CRISPR	Clustered Regularly Interspaced Short Palindromic Repeats
CSF	Cerebrospinal Fluid
CVD	Cardiovascular Diseases
eRNA	Enhancer RNA
GEO	Gene Expression Omnibus
GWAS	Genome Wide Association Studies
HF	Heart Failure
IHF	Ischemic Heart Failure
KLF4	Kruppel Like Factor 4
lncRNA	Long non-coding RNA
MeSH	Medical Subject Headings
MHRT	Myosin Heavy Chain Associated RNA Transcript
miRNA	MicroRNA
ncRNA	Non-coding RNA
OMIM	Online Mendelian Inheritance in Man
ORF	Open Reading Frame
piRNA	Piwi-interacting RNA
RNA	Ribo Nucleic Acid
rRNA	Ribosomal RNA
SLE	Systemic Lupus Erythematosus
SNP	Single Nucleotide Polymorphism
siRNA	Small interfering RNA
snRNA	Small nuclear RNA
snoRNA	Small nucleolar RNA
tRNA	Transfer RNA
WBC	White Blood Cells
